# Continued Chemotherapy After Concurrent Chemoradiotherapy Improves Treatment Outcomes for Unresectable Cutaneous Squamous Cell Carcinoma: An Analysis of 13 Cases

**DOI:** 10.3389/fmed.2019.00207

**Published:** 2019-09-18

**Authors:** Azusa Hiura, Koji Yoshino, Takuya Maeda, Kojiro Nagai, Satoe Oaku, Chisato Yamashita, Megumi Kato, Jiro Uehara, Yasuhiro Fujisawa

**Affiliations:** ^1^Department of Dermatologic Oncology, Tokyo Metropolitan Cancer and Infectious Diseases Center Komagome Hospital, Tokyo, Japan; ^2^Department of Dermatology, University of Tsukuba, Tsukuba, Japan

**Keywords:** concurrent chemoradiotherapy (CCRT), low-dose cisplatin and 5-fluorouracil, overall response rate, OS, disease control rates, 1-year survival rate, continued chemotherapy, unresectable cutaneous squamous cell carcinoma (ucSCC)

## Abstract

**Background:** There is no standard systemic therapy for unresectable cutaneous squamous cell carcinoma (ucSCC), although various chemotherapy regimens have been reported. In our department, concurrent chemoradiotherapy (CCRT) for ucSCC resulted in a 1-year survival rate similar to that of resectable cutaneous squamous cell carcinoma (cSCC). Treatment involves continued chemotherapy after CCRT. Here, we report the importance of continued chemotherapy after CCRT, based on treatment outcomes.

**Patients and Methods:** We retrospectively evaluated 13 patients with ucSCC, assessing the overall survival, overall response rate (ORR), and disease control rate (DCR).

**Results:** CCRT with continued chemotherapy resulted in an ORR of 84.6%, DCR of 92.3%, and 1-year survival rate of 75%. Of the 13 patients treated with CCRT with continued chemotherapy, 6 had no metastasis. The remaining 7 patients developed metastasis to other organs or lymph nodes beyond the regional lymph nodes, although most sites of metastasis were outside the irradiation area.

**Conclusion:** We conclude that CCRT with continued chemotherapy was effective in treating the irradiation site (primary lesion and regional lymph nodes) and any organ metastasis, although, it is unclear for how long the treatment remains effective.

## Introduction

Cutaneous squamous cell carcinoma (cSCC) is the second most common type of non-melanoma skin cancer (1). We consider surgery as an option for treating cSCC during the early stages, but exclude surgical excision as a treatment option for unresectable cSCC (ucSCC) in advanced stages. We define ucSCC as an unresectable case of either the primary site and/or regional lymph nodes (2–6). Currently, there is no standard treatment for ucSCC, although various chemotherapy regimens have been reported.

In our department, concurrent chemoradiotherapy (CCRT) is performed for ucSCC. Chemotherapy and radiotherapy (RT) begin after surgical excision. If either the primary site or regional lymph nodes are unresectable, RT is performed.

We mainly administer chemotherapy regimens of low-dose cisplatin and 5-fluorouracil (low-dose FP) or carboplatin and 5-fluorouracil (FP') (7). In addition, in our department, we continue chemotherapy if the tumor clearly remains at the primary site and/or regional lymph nodes after CCRT.

The treatment outcomes and 1-year survival rates of CCRT for stage IV cSCC in our department are not significantly different from the outcomes for surgical excision cases and unresectable cases with CCRT with continued chemotherapy (8). Here, we report the importance of continued chemotherapy after CCRT.

## Methods

**Figure 1:**
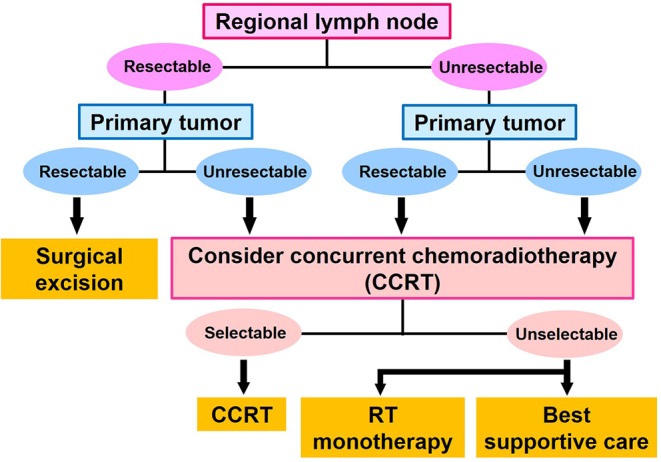
Decision tree for first-line treatment of cutaneous squamous cell carcinoma.

Staging of cSCC was performed using the TNM classification (8th UICC) (9). The first-line treatment for cSCC was determined on the basis of whether the primary site and regional lymph nodes were resectable. If the primary site and regional lymph nodes were resectable, surgical excision was performed. If the primary site and/or regional lymph nodes were not resectable, CCRT was performed. If surgical excision of the regional lymph nodes was difficult, we surgically excised the primary site, and treated the regional lymph nodes with CCRT. If surgical excision of the primary site was difficult, we surgically excised the regional lymph nodes and treated the primary site by CCRT. Patients who had a performance status score ≥3 were not selected for CCRT, and instead underwent RT monotherapy and palliative treatment (Figure 1). No patients underwent chemotherapy monotherapy as a first-line therapy in our department.

The study included 13 patients who were diagnosed with ucSCC and who underwent CCRT with continued chemotherapy. RT irradiation was performed at the primary site and/or regional lymph nodes. The radiation dose was ≥50 Gy. In addition, for chemotherapy during CCRT, we used low-dose FP [(days 1–5) 15 mg/m2 cisplatin plus 800 mg/m2 5-fluorouracil; every 4 weeks] or FP' [(day 1) carboplatin area under the blood concentration-time curve (AUC): 5 (days 1–5) 600 mg/m2 5-fluorouracil; every 4 weeks]. We administered FP' in renal dysfunction cases.

Clinical data included age, sex, primary tumor site, metastasis site, N phase, M phase, histopathological differentiation type, irradiation dose, treatment effect [overall response rate (ORR), disease control rate (DCR)], progression-free survival (PFS), and overall survival (OS). The treatment effect was determined by using computed tomography (CT) every 1 to 3 months based on RECIST (version 1.1) (10) for solid tumors. The PFS and OS were analyzed retrospectively using the Kaplan-Meier method. All statistical analyses were conducted using Microsoft Excel 2016. This study was approved by the Ethics Committee of the Tokyo Metropolitan Cancer and Infectious Disease Center, Komagome Hospital, in accordance with the Declaration of Helsinki. We obtained informed consent from each patient before the treatment.

## Results

**Figure 2:**
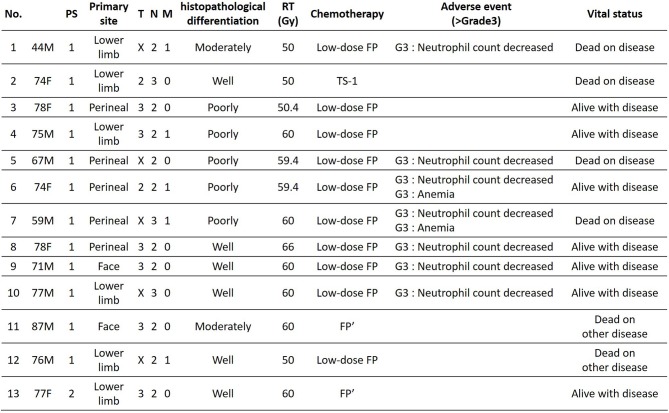
Patients characteristics.

**Figure 3:**
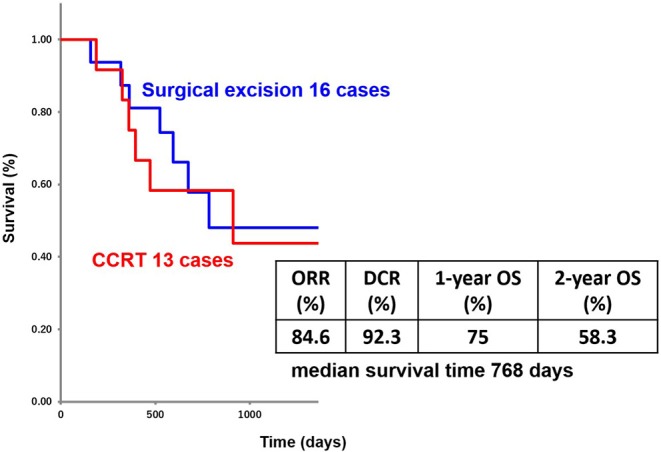
Treatment outcomes of first-line CCRT with continued chemotherapy.

The patients' age ranged from 44 to 87 years (mean age, 72.1 years); the study included 8 men and 5 women. Primary lesions were present in the head and neck in 2 cases, in the lower limbs in 6 cases, and in the perineal region in 5 cases. The clinical stage was 4, and PS was 2 or less for all patients (Figure 2). Analysis for the 13 cases was performed retrospectively using the Kaplan-Meier method. Figure 3 shows the survival curves in the 13 cases according to treatment with CCRT with continued chemotherapy as the first-line therapy for ucSCC. The patients treated with CCRT with continued chemotherapy showed an ORR of 84.6%, DCR of 92.3%, 1-year survival rate of 75 %, 2-year survival rate of 58.3 %, and a median survival time of 768 days.

Of the 13 patients treated with CCRT with continued chemotherapy, 5 patients had lymph node metastases beyond the regional lymph nodes without other organ metastasis before CCRT, and 8 patients had metastases only in the regional lymph nodes before CCRT. Three of 5 patients with lymph node metastases beyond the regional lymph nodes, had no progressive disease during continued chemotherapy, after CCRT. Three of 8 patients with metastases within the regional lymph nodes, had no progressive disease during continued chemotherapy, after CCRT. In 6 patients, who had no progressive disease, there was no difference in histopathological differentiation. Seven patients had lymph node metastasis beyond the regional lymph nodes or other organ metastases during continued chemotherapy (after CCRT), but most sites of metastasis were outside the irradiation area. Seven patients with metastasis during continued chemotherapy showed a 1-year PFS of 64.3 %, and median PFS of 262 days.

In our hospital, 3 patients requested to stop chemotherapy after CCRT, and 1 patient stopped chemotherapy during CCRT due to side effects of chemotherapy. Three patients who were administered low-dose FP stopped chemotherapy after 2, 6, and 8 times, but 6, 10, and 13 months later (respectively), they had recurrence within the irradiation area or other organ metastasis. One patient who stopped due to side effects from chemotherapy had organ metastasis during CCRT.

**Figure 4:**
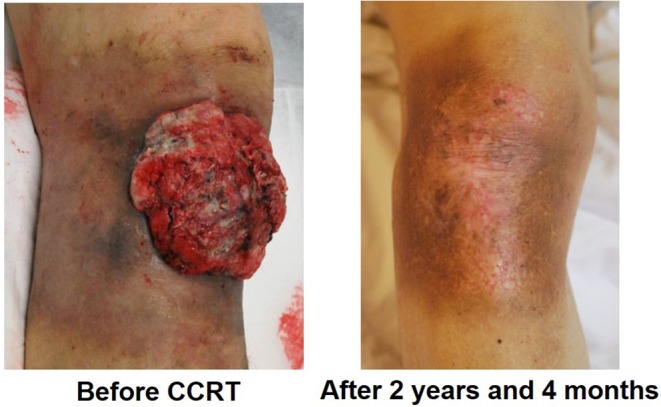
A case of ucSCC with invasion into the right knee ligament.

In our study, among the 13 patients treated with CCRT with continued chemotherapy, 10 patients received low-dose FP therapy, 2 patients received FP' therapy, and 1 patient received other chemotherapy regimens. The results showed low-dose FP and FP' therapy as effective with an ORR of 91.7%, DCR of 91.7%, 1-year survival rate of 72.7%, 2-year survival rate of 63.6%, and median survival time of 804 days. Figure 4 shows an ucSCC with invasion into the right knee ligament and several regional lymph node metastases. It was successfully treated with CCRT with continued chemotherapy. Surgery in this case would have required the patient to undergo an amputation above the knee. Currently, 2 years and 4 months since treatment, the primary lesion has not been observed. The only remnant of the lesion at that location is a scar, moreover, his leg remains and allows the patient to walk normally. Thus, CCRT with continued chemotherapy is a suitable treatment option for ucSCC, as it can improve the quality of life regarding appearance and function.

## Conclusion

Surgical excision is the first choice for the treatment of resectable cSCC, but there is no established treatment for unresectable cases. However, the results of our study show that CCRT with continued chemotherapy was an effective treatment method for unresectable cases. While there are several effective chemotherapy regimens for cSCC, our department mainly uses low-dose FP therapy.

The administration of neoadjuvant chemotherapy with FP therapy significantly improves the overall survival of patients with resectable stage II/III esophageal cancer (11). Moreover, FP therapy is an effective treatment for squamous cell carcinoma.

The age at the onset of cSCC is high, and the mean age of patients in our department was 72.1 years. Cisplatin can cause kidney dysfunction owing to age-related decline in kidney function. Therefore, we believe it is better to use low-dose FP therapy, which enhances the action of 5-fluorouracil as a biochemical modulator with low doses of cisplatin. For patients with renal dysfunction, carboplatin should be used instead of cisplatin.

For most cases of ucSCC after CCRT, the tumor clearly remained at the primary site and/or regional lymph nodes. One patient who stopped chemotherapy during CCRT had an organ metastasis immediately. Three patients who stopped continued chemotherapy after CCRT had recurrence within the irradiation area or other organ metastasis.

Although cutaneous angiosarcoma is a type of skin cancer, patients who received CRT for maintenance chemotherapy showed a significant improvement in OS over patients who received CRT alone (12). From these results, we considered continuing treatment after CCRT using the same chemotherapy regimen.

During continued chemotherapy, 7 of 13 patients had lymph node metastasis beyond the regional lymph nodes or other organ metastasis. Metastasis occurred in 7 patients during continued chemotherapy. This group had a 1-year PFS of 64.3%, and a median PFS of 262 days. Because of these results, we believe that it is important to continue chemotherapy after CCRT. Seven patients who had progressive disease changed to other chemotherapy regimens. Six other patients who had no recurrence within the irradiation range or other organ metastasis continued receiving low-dose FP or FP' therapy.

We recognize it is difficult to control ucSCC with CCRT alone during long-term observation. Thus, although CCRT with continued chemotherapy is effective, metastasis may be observed later. By continuing chemotherapy after CCRT, recurrence within an irradiation area and other organ metastasis were suppressed. Therefore, the treatment outcome of CCRT with continued chemotherapy for ucSCC and that of surgical excision for resectable cSCC were similar (8). Additionally, we understood that continued chemotherapy after CCRT improved the treatment outcome of ucSCC.

In recent years, treatment with immune checkpoint inhibitors has become another option for advanced cSCC. Treatment response for SCC with metastases was 47 and 7% of the patients discontinued the treatment because of an immune-related adverse event (13). Our treatment of CCRT with continued chemotherapy is rarely discontinued due to side effects. We consider chemotherapy as a reasonable treatment option to administer to patients who are elderly. The response rate of CCRT with continued chemotherapy was 84.6%, suggesting that it is an effective treatment for ucSCC.

We evaluated the effectiveness of CCRT with continued chemotherapy for the treatment of ucSCC. We conclude CCRT with continued chemotherapy was effective for treating the irradiation site (primary lesion and regional lymph nodes) and the other organ metastasis.

At this time, due to the small number of cases in this study, the optimal duration of chemotherapy therapy is unknown for patients who receive CCRT with continued chemotherapy without progressive disease. We intend to investigate this in the future.

## Data Availability

All datasets generated for this study are included in the manuscript/supplementary files.

## Ethics Statement

The studies involving human participants were reviewed and approved by the Ethics Committee of the Tokyo Metropolitan Cancer and Infectious Disease Center Komagome Hospital. The patients/participants provided their written informed consent to participate in this study.

## Author Contributions

AH held primary responsibility for communication with the journal and editorial office during the submission process, throughout peer review and during publication. AH was also responsible for ensuring that the submission adheres to all journal requirements including, but not exclusive to, details of authorship, study ethics and ethics approval, clinical trial registration documents and conflict of interest declaration. AH should also be available post-publication to respond to any queries or critiques. All authors contributed conception and design of the study. All authors contributed to manuscript revision, read and approved the submitted version.

### Conflict of Interest Statement

The authors declare that the research was conducted in the absence of any commercial or financial relationships that could be construed as a potential conflict of interest.
